# Two-Stage Super-Efficiency Slacks-Based Model to Assess China’s Ecological Wellbeing

**DOI:** 10.3390/ijerph17197045

**Published:** 2020-09-26

**Authors:** Jundong Hou, Xinxin Ruan, Jun Lv, Haixiang Guo

**Affiliations:** School of Economics & Management, China University of Geosciences, Wuhan 430074, China; ruanxinxin@yeah.net (X.R.); lvjun@cug.edu.cn (J.L.); faterdumk0732@sina.com (H.G.)

**Keywords:** ecological wellbeing performance, super-efficiency SBM model, DEA window analysis method

## Abstract

As industrialization and urbanization in China have significantly increased ecological problems such as environmental pollution and resource waste, it has become important to be able to comprehensively assess ecological wellbeing performance (EWP) when seeking high-quality human wellbeing and economic growth within specific ecological limits. Therefore, to explore the EWP spatial and temporal distribution characteristics, this paper established an evaluation index system that considers ecological economic efficiency and economic welfare efficiency from input and output perspectives. The EWPs in 30 Chinese provinces (autonomous regions, municipalities) from 2006 to 2017 were then measured using a two-stage super-efficiency slacks-based model (Super-SBM) and data envelopment analysis (DEA) window analysis method. It was found that: (1) the average EWP value in the Chinese provinces was relatively low at 0.698, with the highest EWP in Beijing, Hainan, and Shanghai and the lowest in Xinjiang, Ningxia, and Qinghai; (2) the average provincial EWP fluctuated from 2006 to 2017 with a “decline-rise-decline-rise” feature; (3) China’s EWP value was spatially supported by the quadrangular “Beijing-Shanghai-Hainan-Sichuan” pole and continued to radiate to areas along these lines. These research findings provide theoretical insights and practical implications for regional ecological protection and human welfare improvements in China.

## 1. Introduction

Economic globalization and regional economic integration have led to significant increases in China’s urbanization levels from 17.92% in 1978 to 60.60% in 2019 [1]. While this rapid and continuous urbanization has resulted in economic prosperity and social progress [2], it has also resulted in serious environmental problems such as ecological deterioration, environmental pollution, and excessive resource consumption. According to preliminary calculation, in 2019, China’s total energy consumption reached an equivalent of 4.86 billion tons of standard coal, an increase of 3.3% over the previous year [1]. This has not only brought severe challenges to most Chinese provinces [2,3], but has also pushed China’s industrial development to the limits of its resource and environmental carrying capacities [4]. In 2019, China’s carbon dioxide emissions is 9825.8 million tonnes through consumption of oil, gas and coal for combustion related activities, and the growth rate per annum is 3.4%, which accounts for 28.8% of the world’s share [5]. The forecast urbanization and primary energy consumption growth trends for 2050 [6] indicate that these significant ecological pollution and resource depletion pressures are going to continue to be a problem in China [7].

The aim of provincial development is to benefit the residents and ensure a comfortable and satisfactory living environment [8]. However, current industrial and urbanization pressures are far greater than the surrounding ecosystems can bear and economic gains far beyond any increases in human wellbeing [9]. Therefore, to address these environmental and wellbeing issues, ecological consumption efficiency and human wellbeing levels must be attained within ecological thresholds [8]. To realize win-win coordinated economic growth and ecological and human wellbeing aims, it is vital to assess the ecological wellbeing performance (EWP) growth. The EWP was initially proposed by Daly [10] to assess the efficiencies associated with converting natural resources and ecological inputs into human wellbeing [2]. The EWP is essentially a quantitative indicator that reflects the relative changes and degree of harmony between human wellbeing improvements and ecological resource consumption. Consequently, accurately evaluating EWP to properly protect the environment has become a key focus for governments and economic environmental researchers.

Along these lines, this paper evaluated the EWPs in 30 Chinese provinces. Specifically, the main objectives were: (1) to establish an index system to accurately measure the EWP; (2) to design a reasonable model to assess the EWP in 30 Chinese provinces from 2006 to 2017; and (3) to determine the temporal and spatial gaps between the different provinces to enable sustainable policy implementation.

The remainder of this paper is organized as follows. Section 2 reviews related works and the associated limitations and outlines the contributions of this paper; Section 3 details the model specification, variables, and data sources; Section 4 presents the analysis results; and Section 5 concludes the study and gives some potential research directions.

## 2. Literature Review

“Sustainable development” came into use in policy circles in 1987 and was the first overview of the globe considering the environmental features of development from an economic, social, and political comprehensive perspective [11]. Subsequently, this term attracted significant attention in an academic context or that of economics, planning, business, or environmental policy after the first Earth Summit in Rio de Janeiro in 1992 [12]. However, it is argued that the definition of sustainable development was either morally repugnant or logically redundant [13]. Theoretically, ecological economics states that large scale economic growth is restricted by the limits of the environment [10]. Based on this supposition, Daly [10] argued that national sustainability could be evaluated using the ratio of improved human wellbeing to the ecological resources consumed to improve this wellbeing, from which the EWP idea originated. However, as no specific quantitative evaluation indicators or measurement methods were developed, it was not until Rees [14] suggested the ecological footprint per capita as a denominator that the possibility of an EWP measure attracted serious research interest from diverse disciplines such as economics and sociology. This early research was mainly focused on the evaluation approach, indicator design, and the comparison of the EWPs at national, regional, or individual city levels.

There have been two main EWP evaluation methods. The first is a single index ratio method that has generally used ecological footprint resource consumption as the denominator and human wellbeing as the numerator. However, human wellbeing numerator choices have varied: a sustainable wellbeing index [15], years of happiness [16], the human development index (HDI) [17], happy life span [18], happy life index [19], and life expectancy [20], etc. The second EWP evaluation method was a comprehensive index developed using either linear or non-linear approaches, such as regression analysis models, Data Envelopment Analysis (DEA) models [21], and Stochastic Frontier Production models. Table 1 provides a summary of these previous EWP works.

As shown in Table 1, most of these previous studies involved EWP comparisons at national, provincial, or individual city scales using two evaluation methods. Although these studies have significantly contributed to EWP research, there are still some gaps. (1) While there are broadly accepted scale, efficiency, and equity definitions, there has not been any broad consensus on an EWP evaluation index [23]. Therefore, accurate EWP measurements remain to be explored. (2) Existing evaluations have tended to employ proportion analysis methods; however, as countries, provinces, and cities are complex ecological systems with various inputs and outputs, depending solely on proportions cannot fully represent the variations in the ecological resource or human wellbeing levels [8]. Therefore, more recently, Super slack-based measure model (SBM) or stochastic frontier approaches (SFA) have been used to concurrently assess the various input and output efficiencies [2]; however, these methods make it difficult to explain how the key resource and environmental indicators can be improved [34].

Therefore, because of these gaps in previous EWP research, this study makes three main contributions. First, along with the traditional resource consumption ecological input, some related capital indicators are introduced to reflect the ecological capital investment efficiency and more comprehensively compute the provincial level EWP. Second, as EWP growth can be decomposed into the multiplication of two items: economic performance (EP) from the consumption of natural resources up to the ecological threshold and wellbeing performance (WP) from economic growth up to the welfare threshold [9], a super-efficiency network SBM model and DEA window analysis method were combined to examine the ecological input “black box” transformation into a wellbeing output in 30 Chinese provinces. Third, the overall EWP trend and spatial-temporal distribution characteristics were analyzed, the results from which could provide governments with important guidance on promoting better regional EWP in China.

## 3. Methodology

### 3.1. Selection of Input-Output Indicators

**Figure 1:**
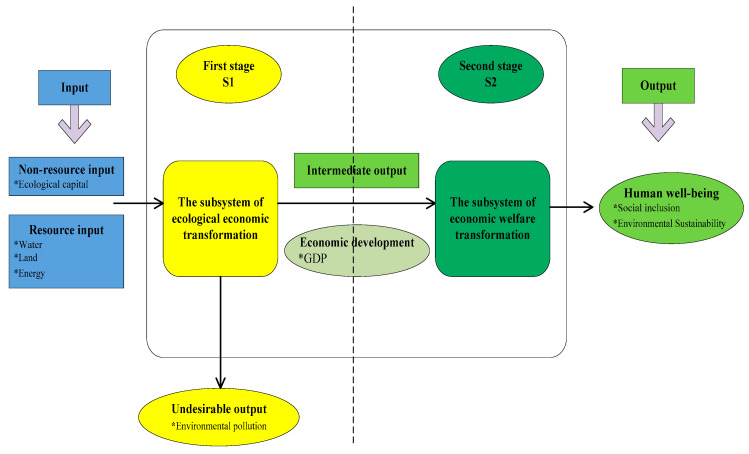
EWP input and output dimensions.

Designing a set of appropriate indicators is the first step to evaluating the EWP. According to the definition of EWP, social benefits and ecological cost can be employed to measure this concept. Nevertheless, the Ecoeconomics theory argues that there are two barriers in green development—one is ecological threshold, which indicates that economic growth is dependent on the supply capacity of natural system resources, and another is welfare threshold, which represents whether economic growth can cause the continuous improvement of social welfare [9]. Based on this proposition, Daly [35] suggested that the EWP could be represented as the economic efficiency of natural expenditure (*EP*) multiplied by the wellbeing performance of economic growth (*WP*), as shown in Formula (1).

(1) $$\begin{array}{ll} EWP & =\frac{WB}{EF}=\frac{GDP}{EF}\times \frac{WB}{GDP}\\ & =EP\times WP \end{array},$$

where *GDP*, *EF*, and *WB*, respectively, represent the economic growth, the ecological footprint, and wellbeing gained. This indicates that the EWP can be divided into two stages (Figure 1): a first stage, focused on the efficiency of transforming the ecological input into economic output (i.e., ecological economy efficiency), and a second stage, focused on the efficiency of converting economic input into wellbeing output (i.e., economic welfare efficiency).

#### 3.1.1. Input Indicators

Essentially, the purpose of the EWP is to obtain maximum welfare output under the least ecological input constraint [36]. As previous studies have indicated that resource consumption was a key ecological input indicator, this paper also selected water, land, and energy consumption as the proxy indicators for ecosystem resource consumption, which were, respectively, indicated by the per capita water consumption, the per capita built-up areas, and the per capita energy consumption. Ecological capital indicators, such as ecological resource capital, ecological environmental capital, and ecological service capital, have also been employed as input indicators [37]. Ecological resource capital is the ecological capital existing in tangible (such as forests, minerals, etc.) and intangible (such as light, heat, water, air, etc.) production resources; because of data availability, this was measured as the ratio of forestry investment to regional GDP. Ecological environmental capital is the environmental ecological capital such as fresh air, clean water, and climate. As the related investment methods are mainly focused on the “three wastes” treatment, ecological environment capital was determined by the ratio of the total environmental pollution treatment investment to GDP. Ecological service capital is related to the construction of urban environmental infrastructure and was measured as the ratio of urban environmental infrastructure construction investment to regional GDP.

#### 3.1.2. Output Indicators

The EWP output indicators included both desirable and undesirable outputs. The undesirable output was the environmental pollution indicator and as environmental pollutants included waste water, exhaust emissions, and waste residues [8], these “three wastes” were selected to represent the environmental pollution indicators. However, unlike previous studies, a composite index was applied to measure these three wastes, as shown in Table 2.

As in previous papers, the core desirable output indicator was GDP [38]. However, as it has been argued that GDP oriented output indicators should be frequently varied, the United Nations Development Program (UNDP) universal human development index (HDI) was chosen. Generally, the HDI measures wellbeing from three specific health, education, and income dimensions [9]. Therefore, based on the sustainable development goals (SDGs) for 2030, the “average education years”, “number of health technicians per 1000 population”, and “urban registered unemployment rate at the end of the year” were taken as the education, medical treatment, and employment levels to comprehensively assess social inclusiveness. The environmental welfare desirable output measure was the forest resource coverage rate. Unlike the traditional GDP economic measurement index, the proportion of R&D investment in GDP was selected as the intermediate rather than a final output index to measure the provincial economic development levels.

Because the Chinese provincial level data does not include the average education years in each province, Equation (2) was used to compute this based on the [30].

(2) $$Average\;education=\frac{6P_{primary\;school}+9\times P_{junior\;middle\;school+12\times P_{high\;school+16\times P_{junior\;college\;or\;above}}}}{P_{primary\;school}+P_{junior\;middle\;school+P_{high\;school+P_{junior\;college\;or\;above}}}}.$$

where *P_i_* is the number of students at each education stage.

### 3.2. Assessment Model

The DEA input-output model first proposed by Charnes et al. [39] has been widely used for efficiency evaluations, including the EWP, for which the indicators included economic development, social welfare, resource consumption, environmental pollution, and others [40]. However, traditional DEA measurements based on radial features have been found to have inaccuracies as they ignore the slack variables [41], fail to take the undesirable outputs into consideration, and can overstate the efficiencies [42]. To overcome these limitations, Tone [43] proposed a slack-based measure model (SBM) that was able to obtain the slack degrees for each input-output indicator in a single-stage DEA efficiency evaluation. However, both the traditional DEA and the improved SBM saw the production processes as a “black box” for single-stage EWP efficiency assessments, which was somewhat unrealistic [34]. Therefore, Tone and Tsutsui [44] extended and proposed a network DEA model within the slacks-based measure framework that was non-radial and was able to individually deal with the inputs/outputs to obtain non-uniform input/output factor efficiencies. Therefore, to comprehensively consider the two stages shown in Figure 1, the super-efficiency DEA model and the SBM model were combined into a super efficiency network SBM model (Super-SBM) with non-radial considerations and undesirable outputs to evaluate the EWP, the development of which is given in the following steps.

(1) Assuming that there are *n* provinces (or decision-making units, DMUs) for the analysis (in this case, *n* = 30), with each province having *m* input indicators (*x*) to produce *v*_1_ desirable outputs ($y^{g}$) and *v*_2_ undesirable outputs ($y^{b}$). We deonte *x_ij_* as a measure of the *ith* input of DMU_j_ and *y_rj_* as the measure of the *rth* output of DMU_j_, where *x_ij_* > 0, *y_ij_* > 0.

(2) Since the orientation of input or output will impact the slack of input or output, in order to ensure the reasonability of computing results, the non-oriented model is applied. Under the condition of variable return to scale, the EWP computation is established as follows:

(3) $$\rho_{se}^{*}=min\frac{\sum_{k=1}^{K}\omega^{k}\left[1+\frac{1}{m_{k}}\left(\sum_{i=1}^{m_{i}}\frac{s_{i}^{k^{-}}}{x_{i0}^{k}}\right)\right]}{\sum_{k=1}^{K}\omega^{k}\left[1-\frac{1}{v_{1k}+v_{2k}}\left(\sum_{r=1}^{v_{1k}}\frac{s_{r}^{gk}}{y_{r0}^{gk}}+\sum_{r=1}^{v_{2k}}\frac{s_{r}^{bk}}{y_{r0}^{bk}}\right)\right]}.$$

In Formula (3), $\rho_{se}^{*}$ is the efficiency value of EWP and ω is the weight. When $\rho_{se}^{*}$ < 1, it indicates that the DMU is inefficient and when $\rho_{se}^{*}$ > 1, the DMU is in an active state.

(4) $$s.t.\{\begin{array}{c} \sum_{j=1,\neq 0}^{n}x_{ij}^{k}\lambda_{j}^{k}+s_{i}^{k^{-}}=\theta^{k}x_{i0}^{k},i=1,\ldots ,m_{k},k=1,\ldots ,K\\ \sum_{j=1,\neq 0}^{n}x_{rj}^{k}\lambda_{j}^{k}+s_{r}^{gk}=\varphi^{k}y_{r0}^{gk},r=1,\ldots ,s_{r},k=1,\ldots ,K\\ \sum_{j=1,\neq 0}^{n}x_{rj}^{k}\lambda_{j}^{k}+s_{r}^{bk}=\delta^{k}y_{r0}^{bk},r=1,\ldots ,s_{r},k=1,\ldots ,K\\ \varepsilon \leq 1-\frac{1}{v_{1k}+v_{2k}}\left(\sum_{r=1}^{v_{1k}}\frac{s_{r}^{gk}}{y_{r0}^{gk}}+\sum_{r=1}^{v_{2k}}\frac{s_{r}^{bk}}{y_{r0}^{bk}}\right)\\ z^{\left(k,h\right)}\lambda^{h}=z^{\left(k,h\right)}\lambda^{k},\sum_{j=1,\neq 0}^{N}\lambda_{j}^{k}=\sum_{k=1}^{K}\omega^{k}=1\\ \lambda_{k}\geq 0,s^{k-}\geq 0,\;s^{gk}\geq 0,\;s^{bk}\geq 0,\;\omega^{k}\geq 0 \end{array}.$$

Formula (4) is the constraint. Where *k* is the number of stages; $m_{k},v_{k}$, $\varphi_{k}$ are the number of inputs, outputs, and intermediate indicators in the *k* stage; (*h*, *k*) denotes the connection from stage *k* to stage *h*; *x*, *y*, and *z* are the input, output, and intermediate output; $\lambda_{k}$ is the weight of stage *k*; and $s^{k-}$, $s^{gk}$, $s^{bk}$ are the slack variables of the input, desirable output, and undesirable output, respectively.

Here, *k* = 2 as there are two stages in this study. As the ecological economy efficiency and welfare transformation efficiency are of equal importance in the EWP evaluation, $\lambda_{1}=\lambda_{2}=0.5$ [34].

(3) DEA Window Analysis. If the time is ignored or the performance of some DMU is compared to a period of time, this can result in an excessive use of resources and production deficiencies in future periods [45]. To overcome these issues, the DEA window analysis described in Charnes et al. [46] can be used to determine the EWP and observe the efficiency shifts over time. The core concept of DEA window analysis suggests that the DMU in each period is a different DMU, which means that different reference sets are selected to evaluate the relative efficiency of the DMU using the moving average method [45]. The moving average method not only increases the number of DMUs, but also allows for the easy comparison of the DMU performance with the DMU performance in other time periods (i.e., longitudinal dimension) or with other DMUs in the same time period (i.e., horizontal dimension), which allows for a comprehensive analysis of the change tendencies in each DMU’s efficiency and a comparison of the differences between the DMUs.

To monitor the flexibility and accuracy of the measurement, a window length needs to be set. Although there is no current theory for defining window length, previous studies have suggested a window of 3–5 years [45]; therefore, as the EWP for the 30 Chinese provinces was assessed over a 12-year period, a 3-year window was selected.

### 3.3. Data Collection

This paper assessed the EWP of Chinese provinces rather than cities for several reasons. First, most data available is at the provincial level. Second, China’s sustainable development goals are focused on provinces, each of which varies in political status and cultural and socio-economic development. Third, the assessment of the provincial EWP provides specific guidance for industrial restructuring and sustainable development [9]. Based on data availability, continuity, and comparability, production decision-making units were selected from 30 Chinese provinces, not including Tibet, Taiwan, Hong Kong, or Macau. Because the Chinese central government proposed to construct a resource-economic, environmentally friendly society that emphasized a harmonious coexistence between man and nature to achieve sustainable development in 2007 and most types of statistics yearbooks failed to be updated to 2019, relevant data from 2006 to 2017 were collected.

The corresponding indicator data shown in Table 2 were acquired from public sources: the annual China Environmental Statistical Yearbooks (2007–2018), China Statistical Yearbooks, China Land and Resources Yearbooks (2007–2018), China Energy Statistical Yearbooks (2007–2018), and China Educational Statistical Yearbooks (2007–2018). Missing data for some certain years or some variables were accounted for using mean substitution and regression imputation. Variables affected by price changes were normalized to 2005 to eliminate the inflation impact and due to the different measurement units, logarithmic methods were adopted to preprocess data such as per capita GDP.

## 4. Empirical Analysis

### 4.1. Overall EWP Analysis

#### 4.1.1. Comprehensive EWP Level

MaxDEA (Version 6.16, Beijing Realworld Software Company Ltd., Beijing, China) software that is most powerful DEA software and supports negative variables and supports the SBM super efficiency model containing the undesirable outputs was used to calculate the EWPs of the 30 Chinese provinces from 2006 to 2017 based on the Super-NSBM model and the DEA window analysis, the results from which are shown in Table 3. Based on the annual average value in each province under the different windows in each year, the 30 provinces were ranked. The overall EWP for the 30 Chinese provinces was 0.698, which was far from SBM-efficient.

**Figure 2:**
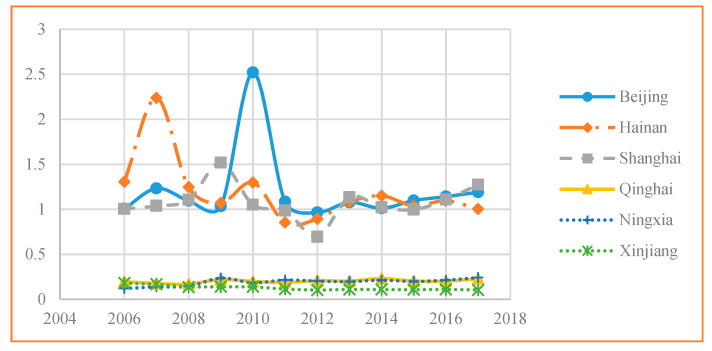
Top and bottom three EWP performers between 2006 and 2017.

The top three and the bottom three EWP performers from 2006 to 2017 are shown in Figure 2. The average EWP in Beijing was the highest, followed by Hainan and Shanghai, all of which were located on the optimal EWP frontier curve. As Beijing was the capital of China and host of the 2008 Olympic Games, prior to and after the Olympic Games in 2008, some high-polluting industries were moved out of the city to Hebei province and several specific eco-environmental protection policies were enforced, which was possibly why Beijing was ranked first. Hainan, which had the second highest EWP, is an island province famous for its eco-tourism industry and therefore, has been paying attention to environmental protection. Shanghai, which had the third highest EWP, underwent industrial restructuring to focus on innovative, high-tech low polluting industries.

However, Xinjiang, Ningxia, and Qinghai had the lowest EWPs (Table 3 and Figure 2) at 0.2022, 0.192, and 0.126, which was similar to the results in [8]. Xinjiang’s EWP was the lowest because of its inefficient natural consumption, Ningxia had poor EWP performance because this province has many heavy chemical industries that consume excessive water resources and cause significant pollution, and Qinghai is dependent on resources and has a fragile ecosystem.

#### 4.1.2. Two Stage Performance

The S1 and S2 EWP stages are shown in Table 4. The average ecological economic transformation efficiency in the 30 Chinese provinces was 0.761 and the average economic welfare transformation efficiency was 0.845; therefore, the S1 efficiency was slightly lower than the S2 efficiency, perhaps because the low ecological economic efficiency led to the low ecological welfare performance. The results in Table 4 indicate that over the entire surveyed period, none of 30 provinces were able to achieve DEA effectiveness concurrently in the two stages. While Beijing, Shanghai, and Tianjin had efficiencies greater than 1 in stage S1, they were less than 1 in stage S2, which indicated that even though these three municipalities achieved coordinated ecological environments and economic growth development, further work is needed to balance human wellbeing and economic growth development. This result was mainly because rapid urbanization and economic development resulted in excessive expansion, which depleted the per capita resources and lowered human wellbeing. However, in contrast, Guangxi, Hainan, Jilin, Inner Mongolia, and Yunnan had efficiencies less than 1 in stage S1, but higher than 1 in stage S2. Most of these provinces are located in the western region and have very fragile ecological environments and are economically backward; therefore, more work is needed to improve their ecological economic transformation performances.

### 4.2. EWP Temporal Analysis

#### 4.2.1. Provincial Perspective

**Figure 3:**
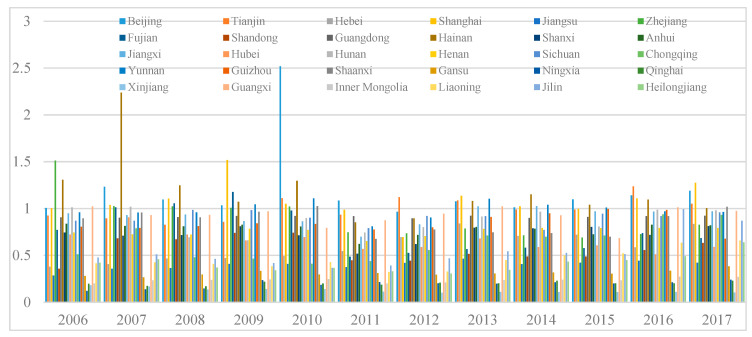
The EWP of the 30 Chinese provinces from 2006 to 2017.

Figure 3 shows the EWP values each year in the 30 Chinese provinces from 2006 to 2017. As can be seen, the overall EWP was not high, with the average EWP being 0.694 in 2006 and only increasing marginally to 0.757 in 2017. The EWP value fluctuated across the 12 years, but in general, was improving, with more noticeable improvements obvious from 2011, the first year of the 12th Chinese Five-year plan in which green sustainable development was first stressed. Specifically, the Chinese provinces began to focus on urbanization quality rather than speed and consequently implemented polices aimed at improving their EWP, such as the Green Development Index, the National New-Type Urbanization Plan, and environmental protection and human wellbeing policies [47].

#### 4.2.2. Regional perspective

To compare the regional EWP differences over time, the 30 provinces were sorted into east, central, west, and northeast regions based on the statistical demarcations of the Chinese Statistical Bureau [48].

**Figure 4:**
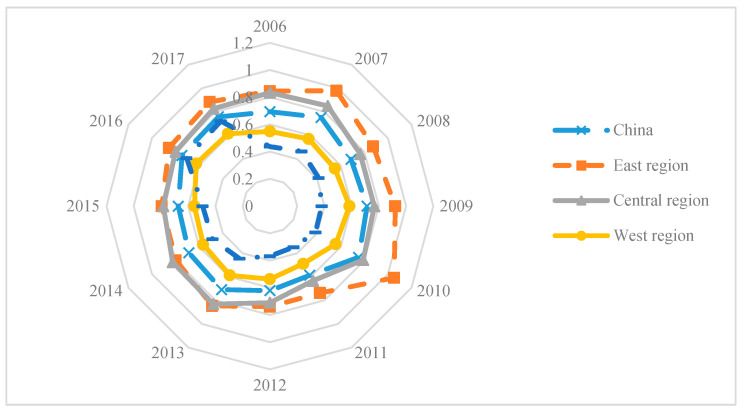
Average EWP values in the different Chinese regions from 2006 to 2017.

Figure 4 shows the average EWPs for the four major regions and the entire country from 2006 and 2017.

As shown in Figure 4, the EWP in all regions increased considerably from 2008 to 2010 and decreased sharply from 2010 to 2011; then, similar to provincial trends, there was a noticeably synchronous improvement in the average EWP from 2011, with the gap between the eastern and the central regions gradually narrowing. Therefore, the temporal EWP distribution trends in the different provinces and in the regions experienced a “decline-rise-decline-rise” variation trend over the period.

### 4.3. EWP Spatial Analysis

#### 4.3.1. EWP Spatial pattern

The average EWP values in the 30 Chinese provinces in the east, central, northeast, and western regions were, respectively, 0.861, 0.785, 0.471, and 0.566, which indicated that there were obvious regional differences between 2006 and 2017 (Figure 4).

As shown in Figure 4, the east and central regions had the highest average EWPs, well above the overall Chinese EWP. The eastern region has natural geographical advantages, preferential policies, and good employment opportunities, which in turn, give rise to positive cumulative economy, education, health, and medical care effects. Further, because of the industrial upgrading in this region, many of the high energy consuming, high polluting industries have been gradually moving to other regions. Although the central region is an important manufacturing agglomeration area and the main region for the high energy consuming, high polluting industrial transfers, the government has implemented several policies, such as the construction of a two-oriented society and the green development of the Yangtze River Economic Belt, to facilitate green industrial clusters. However, in the west and northeast regions, the average EWPs were below the national average, with the northeast region’s being lower than in the west.

Overall, the Chinese provincial EWP pattern was the highest in the eastern region, followed by the central region, the western region, and the northeast region. To some extent, these results reflect the differences in the regional ecological capital, resource consumption, environmental pollution, and residential welfare input-output efficiencies.

**Figure 5:**
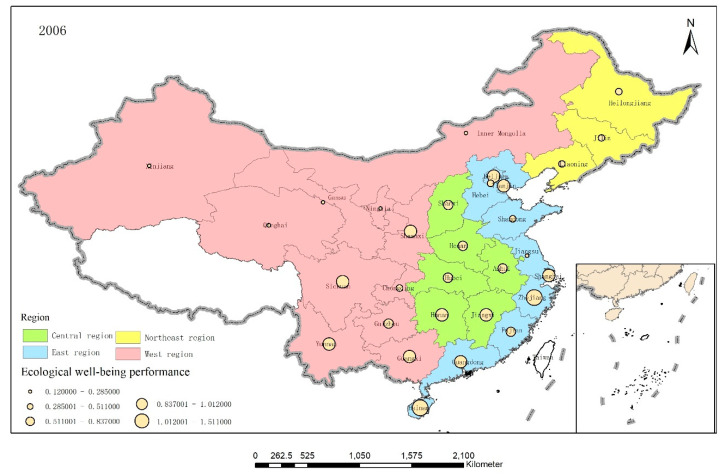
The EWP distributions in the 30 provinces in 2006.

**Figure 6:**
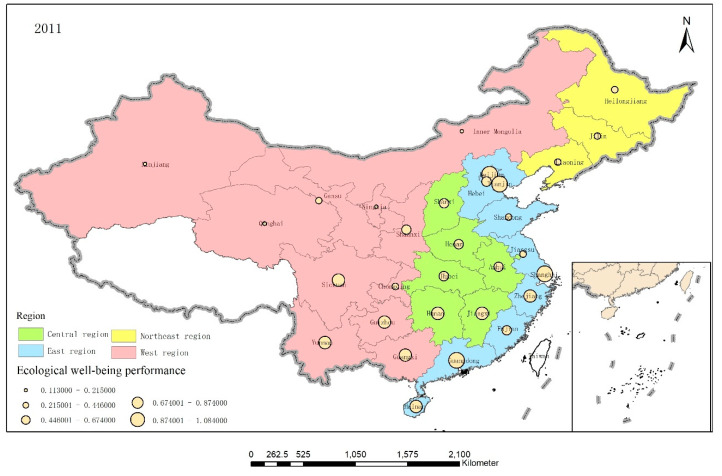
The EWP distributions in the 30 provinces in 2011.

**Figure 7:**
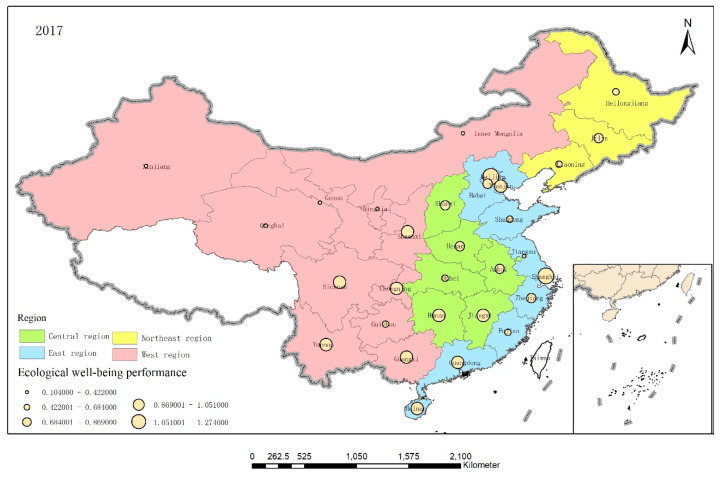
The EWP distributions in the 30 provinces in 2017.

To further analyze the EWP between the different provinces, ArcGIS 10.5 software was used to visualize the EWP distributions in the 30 provinces in 2006, 2011, and 2017, as shown in Figure 5, Figure 6 and Figure 7.

Overall, although the average EWPs in the various regions descended from east to west, the spatial pattern was “high—low—high—low” as the western provinces were pushed to the forefront. China’s EWP value appeared to be spatially supported by a quadrangular pole composed of Beijing, Shanghai, Hainan, and Sichuan and radiated to areas along these lines. However, the central and western regions appeared to form an economic development circle that had low resource utilization efficiency and high pollution while narrowing the economic gap.

#### 4.3.2. Spatial Correlation Analysis

To explore the degree of EWP dependency between the 30 Chinese provinces, global and local spatial autocorrelation statistics measures were utilized [41]. First, the *Moran’s I* statistic was applied, which is the most frequently used measurement for global spatial autocorrelation. The spatial weight matrix was constructed based on the first-order Rook method, after which the spatial correlation test of the EWP in the 30 provinces in China from 2006 to 2017 was conducted. The test results in Table 5 show that in the sample period, the *Moran’s I* index was significant (*p* < 0.05) and the average value was greater than 0.2, indicating that there was a positive spatial autocorrelation of the EWPs between the 30 provinces in China.

Therefore, the EWPs in the 30 Chinese provinces had a certain agglomeration effect, which is the EWP in each province was affected by its own input-output and the EWP and industrial structures of the surrounding provinces. *Moran’s I* statistic is a global statistic that provides greater insight into the local spatial associations between regions; therefore, to visualize the relationship between the regional observation value and its spatial lags, Moran’s scatterplot, which measures the local spatial association, was derived with the EWP of each province on the horizontal axis and the EWP lag term of each province on the vertical axis. The weights were obtained from the spatial weighting matrix.

**Figure 8:**
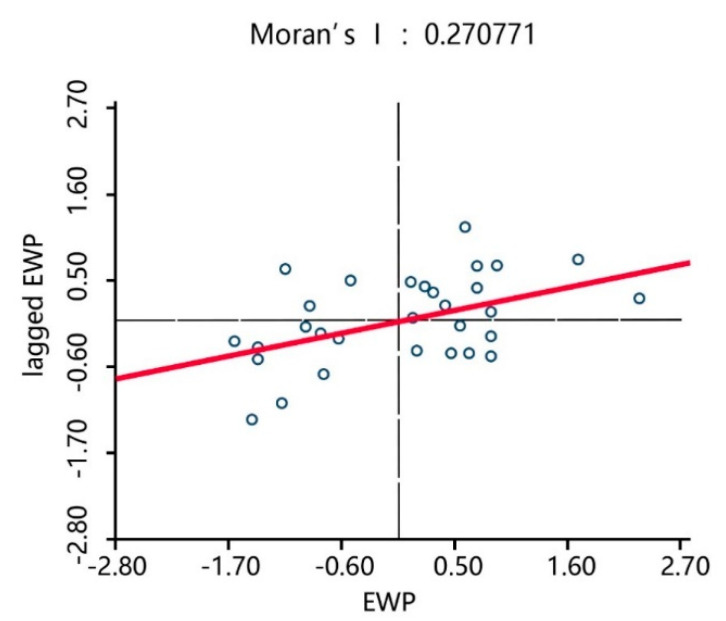
*Moran*’s EWP scatterplots of the 30 provinces in 2006.

**Figure 9:**
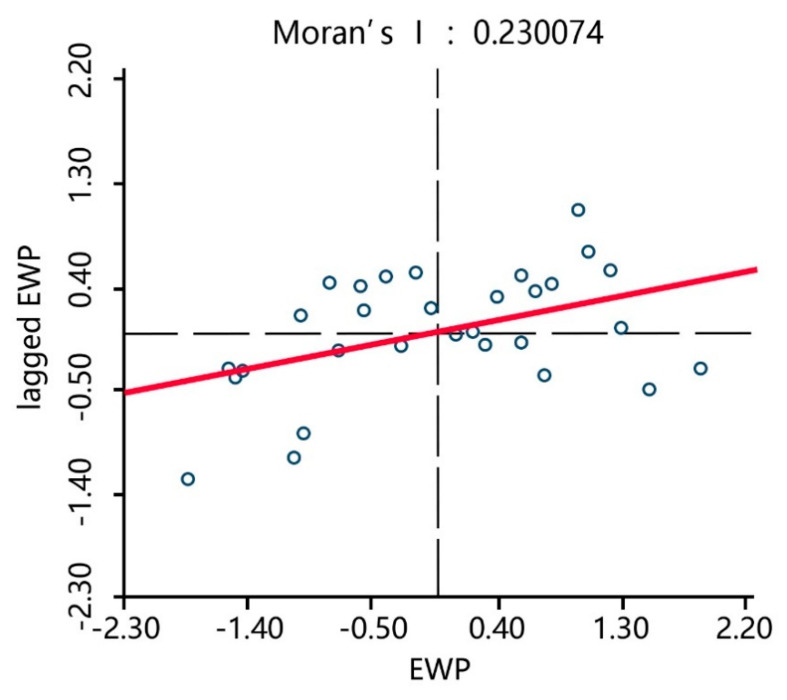
*Moran*’s EWP scatterplots of the 30 provinces in 2011.

**Figure 10:**
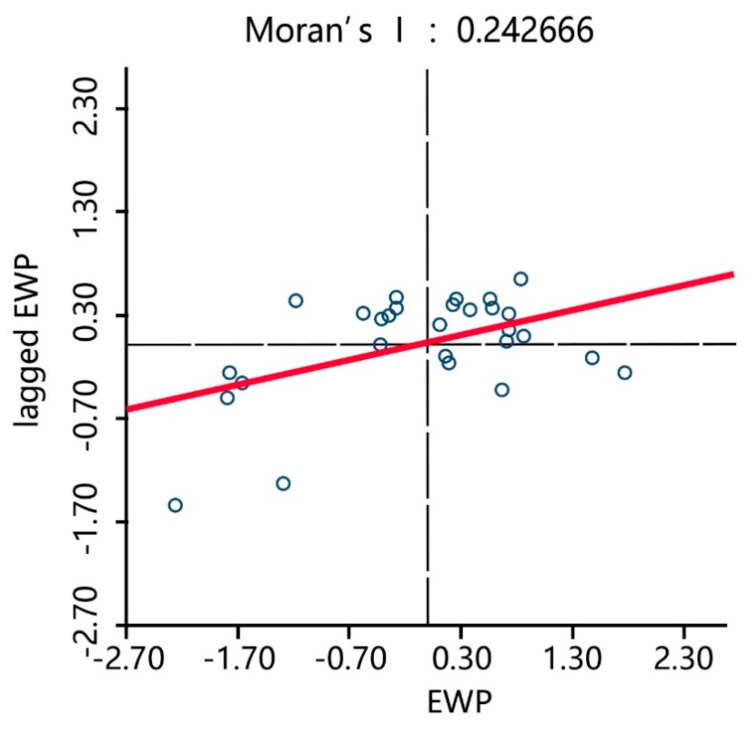
*Moran*’s EWP scatterplots of the 30 provinces in 2017.

Figure 8, Figure 9 and Figure 10 show the Moran’s scatterplots for the Chinese provincial EWPs in 2006, 2011, and 2017, from which it can be seen that most provinces were located in quadrants HH (a province with a high EWP value surrounded by provinces of high values) and LL (a province with a low EWP value surrounded by provinces of low values), indicating that the spatial dependence of the EWP was superior to the spatial heterogeneity. The time trend also indicated that most provinces gradually converged to quadrants HH and LL and that the EWP spatial agglomeration effect in various provinces was increasingly prominent. This result was in line with the previous *Moran’s I* statistics results that identified that the positive EWP spatial autocorrelation was significant between the Chinese provinces.

Therefore, the 30 Chinese provincial EWPs showed a gradient development trend with higher levels in the eastern coastal areas and lower levels in the western areas. As infrastructure construction such as highways and railways has developed, the free flow of element resources has strengthened the communication and cooperation between the regions, with the diffusion effect becoming more obvious over time, which has resulted in the EWPs in the different provinces having a certain spatial correlation with their adjacent regions.

## 5. Conclusions

To estimate regional sustainable development in China’s provinces, it is important to examine their EWP levels. This study designed a comprehensive input-output, two-stage indicator system that concurrently assessed the desirable and undesirable outputs to examine provincial ecological wellbeing. A two-stage super-efficiency network SBM model and DEA analysis window method were employed to measure the EWP in 30 Chinese provinces from 2006 to 2017, from which the following were found.

(1) The average EWP in the 30 Chinese provinces was relatively low in the surveyed period, with the top three performers being Beijing, Hainan, and Shanghai and the poorest being Xinjiang, Ningxia, and Qinghai. While the average EW performances in S1 were slightly lower than in S2, neither were very high, with all 30 provinces failing to achieve concurrent DEA effectiveness in both stages.

(2) Although the overall EWP across China was not high in the sample period, the average level fluctuated every year, but noticeably improved from 2011. Similar to the provincial trends, there was an obvious synchronous improvement in the average EWP in the four major Chinese regions, which indicated that the temporal EWP distribution in the 30 Chinese provinces had a basic “decline-rise-decline-rise” variation trend.

(3) There were significant geographically different features in the EWP in the regional provinces. The eastern Chinese provinces were ranked the highest, followed by the central, west, and northeast regions. The Chinese ecological wellbeing performances were found to be spatially supported by a quadrangular pole composed of Beijing, Shanghai, Hainan, and Sichuan and continued to radiate to areas along these lines. The spatial EWP agglomeration effect in the various provinces in China was more prominent when spatial correlation analysis methods were employed.

This paper makes three main contributions at least. First, although some works used province-level data to examine the EWP in China, most of their results focused on the single stage efficiency value, however our paper decomposed the EWP into the multiplication of EP and WP and further obtained two-stage super-efficiency performances. Second, some related capital indicators were innovatively introduced to reflect the ecological capital investment efficiency, which can be beneficial for comprehensively understating the provincial level EWP. Third, although some studies applied statistical methods to evaluate the EWP, their results were not considered from a spatial perspective. To overcome this limitation, we used spatial-temporal approaches to analyze and concluded the valuable spatial-temporal distribution rules. Even so, there were also some limitations. First, China’s provincial regions need to be categorized based on their different levels to further enhance the generality of the findings regarding the spatial-temporal EWP differentiation. The ecological wellbeing performances of the individual provinces also need to be assessed as they have a duty to improve their EWP and experience sharing, organizational learning, and policy diffusion across cities can advance urban sustainable development. Besides, our results may be less accurate than city-level research because of ignoring the heterogeneity in each province; therefore, in the future, we can use individual city’s data to depict more informative and reliable results. Second, the link between EWP and some factors in China’s development should be studied to determine the EWP driving mechanisms and influence determinants, such as the relationships between industrial agglomeration, green technology development, and EWP.

## Figures and Tables

**Table 1:** Related ecological wellbeing performance (EWP) studies and the varying scales.

Scale	Authors	Method	Objective Area	Time Period
National level	Common (2007) [18]	The ratio of happiness adjusted life expectancy for the average individual to per capita energy consumption	75 nations	1995–2005
	Moran et al. (2008) [22]	The ratio of human development index (HDI) to the ecological footprint	93 countries	1975–2003
	Dietz et al. (2009) [23]	A Stochastic Frontier Production model	135 countries	1999
	Knight and Rosa (2011) [24]	Regress the average life satisfaction and the ecological footprint per capita	105 countries	2005
	Dietz et al. (2012) [20]	The ratio of environmental stress to wellbeing	58 nations	1961–2003
	Jorgenson et al. (2014) [25]	The adjusted ratio of per capita energy consumption to average life expectancy	12 European countries	1992–2010
	Zhu et al. (2015) [26]	The ratio of HDI to ecological footprint	G20 countries	1995–2008
	Zhang et al. (2018) [27]	The ratio of HDI to per capita normalized ecological footprint	82 countries	2012
Regional level	Feng and Yuan (2016) [28]	The ratio of HDI to normalized per capita ecological footprint	30 provinces in China	2005–2010
	Xu et al. (2017) [29]	Exploratory Spatial Data Analysis method	30 provinces in China	2005–2014
	Xiao and Zhang (2019) [30]	Improved Stochastic Frontier Analysis model	30 provinces of China	2004–2015
	Fang and Xiao (2019) [31]	Super-DEA	30 provinces of China	2005–2016
	Feng et al. (2019) [9]	The ratio of HDI to Ecological Footprint	30 provinces of China	1994–2014
Individual cities	He and Chen (2011) [32]	The ratio of HDI to per capita ecological footprint index	Shannxi province	1990–2009
	Long and Wang (2017) [33]	Super-slack-based measure (Super-SBM)	Shanghai city	2006–2014
	Long (2019) [34]	Super-efficiency network SBM model	35 major cities in China	2011–2015
	Bian, Ren & Liu (2020) [8]	Super-SBM	30 provincial capital cities in China	2011–2016
	Bian et al. (2020) [2]	Super-SBM	278 Chinese cities	2005–2016

**Table 2:** Main characteristics for the EWP input-output indicators.

Category	1st Tier Indicators	2nd Tier Indicators	3rd Tier Indicators
Input Indicators	Ecological capital	Ecological service capital	Ratio of investment in urban environmental infrastructure construction to regional GDP
		Ecological environment capital	Ratio of total investment in environmental pollution control to GDP
		Ecological resource capital	Ratio of forestry investment completed in the current year to regional GDP
	Consumption of ecological resources	Energy consumption	Per capita energy consumption (tonnes of standard coal/person)
		Land resource consumption	Per capita builtup area (km^2^/10,000 people)
		Water resource consumption	Per capita water consumption (m^3^/person)
Undesirable output	Environmental pollution	Wastewater discharge	Per capita chemical oxygen demand (tonnes/person)
			Per capita ammonia nitrogen emissions (tonnes/person)
		Exhaust gas discharge	Per capita sulfur dioxide emissions (tonnes/person)
			Per capita smoke (powder) dust emission (tonnes/person)
		Solid waste discharge	Per capita production of industrial solid waste (tonnes/person)
			Per capita amount of municipal solid waste (tonnes/person)
Desirable Output	Economic development	Technological innovation	R&D input as a proportion of GDP
	Social welfare	Social Inclusion	Urban registered unemployment rate at the end of the year
		Environmental Sustainability	Average education (years)
			Number of health technicians per 1000 population
			Forest coverage

**Table 3:** The EWPs of 30 provinces during 2006–2017.

Region	Provinces	2006	2007	2008	2009	2010	2011	2012	2013	2014	2015	2016	2017	Average	Rank
East region	Beijing	1.006	1.232	1.096	1.034	2.519	1.084	0.965	1.077	1.012	1.097	1.141	1.190	1.204	1
	Tianjin	0.925	0.894	0.826	0.857	1.110	0.934	1.120	1.086	0.988	0.989	1.237	1.051	1.002	4
	Hebei	0.379	0.408	0.465	0.471	0.492	0.544	0.694	0.840	0.708	0.720	0.585	0.834	0.595	20
	Shanghai	1.004	1.038	1.106	1.518	1.051	0.986	0.694	1.135	1.022	0.997	1.108	1.274	1.078	3
	Jiangsu	0.285	0.358	0.365	0.409	0.409	0.376	0.420	0.463	0.406	0.421	0.443	0.422	0.398	25
	Zhejiang	1.511	1.026	1.023	1.008	1.020	0.745	0.734	0.785	0.713	0.690	0.726	0.827	0.901	10
	Fujian	0.772	1.013	1.054	1.177	0.977	0.486	0.526	0.566	0.582	0.577	0.736	0.684	0.763	15
	Shandong	0.358	0.682	0.670	0.740	0.741	0.446	0.443	0.514	0.487	0.488	0.555	0.632	0.563	21
	Guangdong	0.906	0.901	0.907	0.921	0.921	0.918	0.895	0.923	0.900	0.909	0.919	0.922	0.912	8
	Hainan	1.306	2.237	1.246	1.071	1.297	0.853	0.894	1.081	1.150	1.039	1.095	1.003	1.189	2
Central region	Shanxi	0.743	0.709	0.716	0.809	0.713	0.518	0.620	0.795	0.789	0.802	0.718	0.811	0.729	17
	Anhui	0.837	0.813	0.809	0.826	0.807	0.622	0.718	0.802	0.783	0.725	0.826	0.819	0.782	14
	Jiangxi	0.949	0.929	0.935	0.865	0.862	0.698	0.831	1.022	1.026	0.969	0.968	0.970	0.919	7
	Hubei	0.722	0.906	0.722	0.658	0.695	0.569	0.587	0.680	0.587	0.606	0.511	0.590	0.653	18
	Hunan	1.012	1.018	0.691	0.661	0.897	0.744	0.800	0.913	0.965	0.807	0.986	0.981	0.873	11
	Henan	0.743	0.724	0.722	0.784	0.770	0.651	0.705	0.781	0.792	0.790	0.792	0.792	0.754	16
West region	Sichuan	0.869	0.869	0.985	0.982	0.901	0.790	0.921	0.919	0.772	0.943	0.919	0.959	0.902	9
	Chongqing	0.511	0.788	0.479	0.463	0.411	0.438	0.555	0.712	0.698	0.712	0.938	0.932	0.637	19
	Yunnan	0.958	0.956	0.959	1.044	1.108	0.809	0.904	1.104	1.039	1.010	0.970	0.963	0.985	5
	Guizhou	0.805	0.793	0.812	0.841	0.834	0.776	0.798	0.909	0.948	0.995	0.985	0.678	0.848	13
	Shaanxi	0.894	0.956	0.905	0.965	1.024	0.674	0.775	0.745	0.736	0.699	0.918	1.018	0.859	12
	Gansu	0.281	0.266	0.296	0.334	0.296	0.312	0.293	0.307	0.318	0.305	0.336	0.384	0.311	26
	Ningxia	0.120	0.138	0.146	0.236	0.184	0.215	0.202	0.197	0.212	0.199	0.213	0.241	0.192	29
	Qinghai	0.197	0.175	0.171	0.220	0.199	0.188	0.208	0.203	0.230	0.203	0.203	0.229	0.202	28
	Xinjiang	0.180	0.168	0.136	0.140	0.140	0.113	0.102	0.110	0.109	0.108	0.110	0.104	0.126	30
	Guangxi	1.023	0.928	0.931	0.970	0.793	0.874	0.943	1.023	0.927	0.683	1.012	0.974	0.923	6
	Inner Mongolia	0.201	0.235	0.238	0.243	0.247	0.200	0.210	0.237	0.241	0.235	0.272	0.271	0.236	27
Northeast region	Liaoning	0.415	0.427	0.410	0.384	0.428	0.324	0.329	0.449	0.499	0.521	0.634	0.657	0.456	23
	Jilin	0.476	0.511	0.462	0.418	0.366	0.393	0.467	0.543	0.526	0.512	0.993	0.869	0.545	22
	Heilongjiang	0.421	0.458	0.371	0.341	0.367	0.331	0.307	0.346	0.432	0.449	0.490	0.638	0.412	24
China		0.694	0.752	0.688	0.713	0.753	0.587	0.622	0.709	0.687	0.673	0.745	0.757	0.698	
East region		0.845	0.979	0.876	0.921	1.054	0.737	0.739	0.847	0.797	0.793	0.855	0.884	0.861	
Central region		0.834	0.850	0.766	0.767	0.791	0.634	0.710	0.832	0.824	0.783	0.800	0.827	0.785	
West region		0.549	0.570	0.551	0.585	0.558	0.490	0.537	0.588	0.566	0.554	0.625	0.614	0.566	
Northeast region		0.437	0.465	0.414	0.381	0.387	0.349	0.368	0.446	0.486	0.494	0.706	0.721	0.471	

**Table 4:** The EWPs in stage S1 and S2 from 2006 to 2017.

Region		2006		2007		2008		2009		2010		2011		2012		2013		2014		2015		2016		2017		2006–2017	
		S1	S2	S1	S2	S1	S2	S1	S2	S1	S2	S1	S2	S1	S2	S1	S2	S1	S2	S1	S2	S1	S2	S1	S2	S1	S2
East region	Beijing	1.013	0.959	1.491	0.233	1.164	0.788	1.028	1.010	4.588	0.395	1.129	0.816	1.024	0.883	1.056	1.043	1.026	0.960	1.180	0.773	1.238	0.671	1.274	1.016	1.434	0.796
	Tianjin	1.000	0.861	1.000	0.809	1.000	0.706	0.941	0.804	1.152	0.867	0.925	1.010	1.123	0.997	1.108	0.956	1.020	0.922	1.031	0.890	1.336	0.591	1.077	1.001	1.059	0.868
	Hebei	0.393	0.892	0.457	0.753	0.535	0.747	0.515	0.860	0.557	0.799	0.600	0.835	0.805	0.768	1.000	0.725	0.816	0.707	0.854	0.654	0.648	0.481	1.000	0.715	0.682	0.745
	Shanghai	1.002	1.000	1.110	0.651	1.099	0.771	1.310	1.494	1.099	0.752	1.013	0.903	1.000	0.532	1.133	1.028	1.069	0.904	1.037	0.923	1.199	0.534	1.451	0.800	1.127	0.858
	Jiangsu	0.451	0.328	0.607	0.340	0.610	0.327	0.596	0.355	0.603	0.329	0.540	0.302	0.656	0.359	0.662	0.429	0.542	0.467	0.546	0.492	0.570	0.371	0.550	0.540	0.578	0.387
	Zhejiang	1.602	0.746	1.119	0.746	1.049	0.942	1.024	0.942	1.040	0.944	0.717	0.983	0.741	0.927	0.768	1.030	0.749	0.809	0.663	0.963	0.712	0.651	0.807	1.000	0.916	0.890
	Fujian	0.724	1.000	1.022	0.990	1.085	0.934	1.165	1.099	0.933	1.163	0.461	1.000	0.505	0.983	0.543	1.000	0.548	0.984	0.536	1.002	0.706	0.667	0.649	1.000	0.740	0.985
	Shandong	0.469	0.464	1.000	0.517	0.954	0.552	1.000	0.587	1.000	0.588	0.582	0.462	0.598	0.430	0.700	0.461	0.647	0.364	0.608	0.497	0.719	0.334	0.827	0.554	0.759	0.484
	Guangdong	1.000	0.827	1.000	0.820	1.000	0.830	1.000	0.854	1.000	0.853	1.000	0.848	1.000	0.809	1.000	0.856	1.000	0.819	1.000	0.834	1.000	0.566	1.000	0.856	1.000	0.814
	Hainan	0.669	4.626	0.964	3.468	1.231	1.320	0.983	1.350	1.297	1.290	0.757	1.583	0.848	1.351	1.030	1.308	0.674	3.850	0.845	1.988	0.717	2.091	0.915	1.389	0.911	2.134
Central region	Shanxi	1.000	0.591	1.000	0.549	1.000	0.558	1.000	0.679	0.931	0.570	0.634	0.779	0.818	0.676	1.000	0.660	1.000	0.651	1.000	0.670	0.863	0.456	1.000	0.682	0.937	0.627
	Anhui	1.000	0.719	1.000	0.686	1.000	0.679	1.000	0.704	1.000	0.676	0.744	0.614	0.863	0.661	1.000	0.669	0.964	0.622	0.925	0.503	1.000	0.469	1.000	0.693	0.958	0.641
	Jiangxi	1.000	0.903	1.000	0.867	1.000	0.878	0.947	0.763	0.931	0.816	0.708	0.944	0.840	1.053	0.979	1.179	1.031	1.010	1.000	0.941	1.000	0.625	1.000	0.942	0.953	0.910
	Hubei	0.756	0.683	1.000	0.829	0.797	0.728	0.714	0.761	0.790	0.602	0.615	0.623	0.646	0.641	0.751	0.695	0.629	0.619	0.634	0.749	0.560	0.372	0.631	0.851	0.710	0.679
	Hunan	1.015	0.980	1.029	0.992	0.691	0.944	0.674	0.820	0.941	0.837	0.768	0.803	0.809	0.972	0.957	0.871	1.000	0.932	0.839	0.759	1.000	0.649	1.000	0.963	0.894	0.877
	Henan	1.000	0.591	1.000	0.567	1.000	0.565	1.000	0.645	1.000	0.626	0.799	0.629	0.886	0.627	1.000	0.641	1.000	0.655	1.000	0.653	1.000	0.437	1.000	0.655	0.974	0.608
West region	Sichuan	1.000	0.768	1.000	0.768	1.066	0.769	1.029	0.926	1.000	0.820	0.879	0.654	1.000	0.854	1.000	0.851	0.838	0.716	1.000	0.892	0.974	0.565	1.000	0.922	0.982	0.792
	Chongqing	0.622	0.414	1.000	0.651	0.631	0.414	0.542	0.536	0.466	0.569	0.493	0.639	0.615	0.740	0.798	0.780	0.669	1.001	0.725	0.820	1.000	0.589	1.000	0.873	0.713	0.669
	Yunnan	1.000	0.919	1.000	0.916	1.000	0.922	1.024	1.098	1.125	1.105	0.774	1.088	0.875	1.107	1.102	1.027	0.989	1.221	1.005	1.044	1.000	0.628	1.000	0.929	0.991	1.000
	Guizhou	1.000	0.673	1.000	0.658	1.000	0.683	1.000	0.726	1.000	0.716	0.894	0.760	0.881	0.845	1.000	0.833	1.000	0.901	0.978	1.132	1.000	0.646	0.621	1.426	0.948	0.833
	Shaanxi	1.000	0.808	1.116	0.552	1.000	0.826	1.056	0.751	1.118	0.665	0.711	0.632	0.818	0.801	0.797	0.644	0.756	0.729	0.696	0.737	0.955	0.533	1.110	0.626	0.928	0.692
	Gansu	0.527	0.230	0.537	0.201	0.589	0.194	0.544	0.273	0.478	0.332	0.463	0.390	0.447	0.447	0.477	0.408	0.466	0.467	0.449	0.427	0.507	0.296	0.580	0.462	0.505	0.344
	Ningxia	0.158	0.529	0.183	0.449	0.190	0.549	0.221	1.050	0.183	0.901	0.204	0.940	0.226	0.806	0.214	0.882	0.216	1.108	0.193	1.256	0.211	0.751	0.195	1.905	0.199	0.927
	Qinghai	0.351	0.321	0.340	0.311	0.313	0.425	0.351	0.569	0.358	0.385	0.330	0.420	0.395	0.361	0.332	0.523	0.360	0.572	0.285	0.739	0.292	0.445	0.306	0.881	0.334	0.496
	Xinjiang	0.239	0.975	0.267	0.694	0.227	0.632	0.189	0.929	0.225	0.639	0.174	0.722	0.157	0.743	0.165	0.778	0.162	0.788	0.160	0.782	0.172	0.495	0.173	0.555	0.193	0.728
	Guangxi	0.806	2.080	1.000	0.866	1.000	0.871	1.000	0.942	0.781	1.002	0.897	0.895	0.940	1.017	0.975	1.224	0.919	1.053	0.625	1.349	0.876	1.083	1.000	0.950	0.901	1.111
	Inner Mongolia	0.163	1.763	0.203	1.443	0.211	1.368	0.205	1.510	0.223	1.241	0.176	1.417	0.188	1.318	0.212	1.277	0.218	1.250	0.212	1.240	0.244	0.880	0.239	1.466	0.208	1.348
Northeast region	Liaoning	0.374	1.000	0.405	0.777	0.404	0.537	0.372	0.639	0.416	0.620	0.314	0.605	0.325	0.590	0.430	0.862	0.457	1.095	0.414	1.875	0.563	0.862	0.618	1.013	0.424	0.873
	Jilin	0.445	0.975	0.503	0.885	0.429	0.859	0.377	1.028	0.346	0.805	0.354	1.007	0.397	1.481	0.435	1.886	0.409	2.038	0.406	1.896	0.885	1.088	0.811	1.325	0.483	1.273
	Heilongjiang	0.381	1.000	0.429	1.003	0.342	0.974	0.310	1.000	0.334	1.000	0.302	0.876	0.278	0.947	0.312	0.991	0.388	1.000	0.405	1.000	0.448	0.743	0.610	1.000	0.378	0.961
China		0.739	0.954	0.826	0.800	0.787	0.743	0.770	0.857	0.897	0.764	0.632	0.806	0.690	0.824	0.765	0.884	0.719	0.974	0.708	0.949	0.780	0.652	0.815	0.933	0.761	0.845
East region		0.832	1.170	0.977	0.933	0.973	0.792	0.956	0.936	1.327	0.798	0.772	0.874	0.830	0.804	0.900	0.884	0.809	1.079	0.830	0.902	0.885	0.696	0.955	0.887	0.921	0.896
Central region		0.962	0.745	1.005	0.748	0.915	0.725	0.889	0.729	0.932	0.688	0.711	0.732	0.810	0.772	0.948	0.786	0.937	0.748	0.900	0.713	0.904	0.501	0.939	0.798	0.904	0.724
West region		0.624	0.862	0.695	0.683	0.657	0.696	0.651	0.846	0.632	0.761	0.545	0.778	0.595	0.822	0.643	0.839	0.599	0.891	0.575	0.947	0.657	0.628	0.657	1.000	0.627	0.813
Northeast region		0.400	0.992	0.446	0.888	0.392	0.790	0.353	0.889	0.365	0.808	0.323	0.829	0.333	1.006	0.392	1.246	0.418	1.378	0.408	1.590	0.632	0.898	0.680	1.113	0.428	1.036

Note: S1 and S2 represents the first and second stage of EWP, i.e., the ecological economic efficiency and economic welfare efficiency, respectively.

**Table 5:** *Moran’s I* for China’s Provincial EWP from 2006 to 2017.

Year	*Moran’s I*	*p*	Year	*Moran’s I*	*p*
2006	0.2707	0.0070	2012	0.3245	0.0050
2007	0.2559	0.0060	2013	0.3086	0.0030
2008	0.2764	0.0080	2014	0.3144	0.0050
2009	0.1883	0.0340	2015	0.2545	0.0090
2010	0.1736	0.0134	2016	0.2694	0.0100
2011	0.2301	0.0170	2017	0.2427	0.0150
